# Lipid-Rich Extract from Mexican Avocado Seed (*Persea americana* var. drymifolia) Reduces *Staphylococcus aureus* Internalization and Regulates Innate Immune Response in Bovine Mammary Epithelial Cells

**DOI:** 10.1155/2019/7083491

**Published:** 2019-09-12

**Authors:** Marisol Báez-Magaña, Alejandra Ochoa-Zarzosa, Nayeli Alva-Murillo, Rafael Salgado-Garciglia, Joel Edmundo López-Meza

**Affiliations:** ^1^Centro Multidisciplinario de Estudios en Biotecnología-FMVZ, Universidad Michoacana de San Nicolás de Hidalgo, Km 9.5 Carretera Morelia-Zinapécuaro, Posta Veterinaria C.P., 58893 Morelia, Michoacán, Mexico; ^2^Departamento de Biología, División de Ciencias Naturales y Exactas, Universidad de Guanajuato, Guanajuato, Mexico; ^3^Instituto de Investigaciones Químico Biológicas, UMSNH. Ciudad Universitaria, Morelia, Michoacán, Mexico

## Abstract

Bovine mammary epithelial cells (bMECs) are capable of initiating an innate immune response (IIR) to invading bacteria. *Staphylococcus aureus* is not classically an intracellular pathogen, although it has been shown to be internalized into bMECs. *S. aureus* internalizes into nonprofessional phagocytes, which allows the evasion of the IIR and turns antimicrobial therapy unsuccessful. An alternative treatment to control this pathogen is the modulation of the innate immune response of the host. The Mexican avocado (*Persea americana* var. drymifolia) is a source of molecules with anti-inflammatory and immunomodulatory properties. Hence, we analyze the effect of a lipid-rich extract from avocado seed (LEAS) on *S. aureus* internalization into bMECs and their innate immunity response. The effects of LEAS (1-500 ng/ml) on the *S. aureus* growth and bMEC viability were assessed by turbidimetry and MTT assays, respectively. LEAS did not show neither antimicrobial nor cytotoxic effects. *S. aureus* internalization into bMECs was analyzed by gentamicin protection assays. Interestingly, LEAS (1-200 ng/ml) decreased bacterial internalization (60-80%) into bMECs. This effect correlated with NO production and the induction of the gene expression of IL-10, while the expression of the proinflammatory cytokine TNF-*α* was reduced. These effects could be related to the inhibition of MAPK p38 (∼60%) activation by LEAS. In conclusion, our results showed that LEAS inhibits the *S. aureus* internalization into bMECs and modulates the IIR, which indicates that avocado is a source of metabolites for control of mastitis pathogens.

## 1. Introduction

The innate immune response (IIR) is the first line of defense of organisms, which has a relevant role in the protection against pathogens. The participation of professional phagocytic cells (c.a. macrophages, dendritic cells, and circulating leukocytes) in the IIR is fundamental; however, nonprofessional phagocytic cells (c.a. epithelium, endothelium, osteoblast, and fibroblast cells) also have a relevant role [1]. In this sense, bovine mammary epithelial cells (bMECs) play an important role in the IIR of the mammary gland acting as a physical barrier and as initial sensors of danger with the capacity to mount a defense response [2]. The IIR regulation by immunomodulatory molecules such as fatty acids and vitamins has been widely demonstrated and involves epigenetics changes that can be stably maintained or adapted to changing environments [3, 4]. For the above, the search for modulators that improve the bMEC IIR increases the opportunity to identify novel therapeutics.

*S. aureus* is the main pathogen responsible for subclinical bovine mastitis, a chronic and recurrent disease that affect dairy cattle worldwide [5, 6]. This bacterium has the ability to be internalized into the cells, which allows it to evade the IIR of the host; this characteristic has been associated with the recurrence of mastitis [7, 8]. In previous reports, we showed that immunomodulatory molecules (short and medium chain fatty acids, and cholecalciferol) inhibit *S. aureus* internalization into bMECs regulating the IIR, suggesting an immunomodulatory role in host-pathogen interaction [3, 8, 9].

For a long time, plants have been a rich source of antibacterial, antiviral, and immunomodulatory metabolites. In this sense, avocado (*Persea americana*) is a very nutritious fruit (rich in saturated and unsaturated fatty acids) that possesses different compounds with antioxidant, anticancer, antimicrobial, and anti-inflammatory properties [10]. Diverse reports have shown that avocado metabolites have immunomodulatory properties. For example, an avocado/soybean unsaponifiable (ASU) mixture decreased the expression of TNF-*α*, IL-1*β*, COX-2, and iNOS in bovine chondrocytes and human monocyte/macrophages [11, 12]. Likewise, seven-carbon sugars typical of avocado (mannoheptulose and perseitol) inhibited *Malassezia furfur* internalization into human keratinocytes and induced expression of the antimicrobial peptide HBD-2 [13]. Also, avocado aliphatic acetogenins (lipid derivative molecules present in mesocarp and endocarp) favored the apoptosis induction and cell cycle arrest in different human cancer cell lines [14, 15]. Furthermore, the anti-inflammatory and photoprotective effects of polyhydroxylated fatty alcohols (synonymous of acetogenins) extracted from *Persea gratissima* on human keratinocytes damaged by UV have been demonstrated [16]. However, it is unknown if lipid derivative molecules from Mexican avocado seed (*Persea americana* var. drymifolia) display immunomodulatory properties in host-pathogen interaction. Thus, in this work, we analyzed the effects of a lipid-rich extract from Mexican avocado seed (LEAS) on *S. aureus* internalization into bMECs. In addition, the effects of LEAS on the IIR of bMECs were analyzed.

## 2. Materials and Methods

### 2.1. Lipid-Rich Extract from Avocado Seed (LEAS)

Mexican avocado fruits (*Persea americana* var. drymifolia) used in this study were collected when physiological maturity was achieved. The LEAS was obtained according to Rosenblat et al. [16]. Briefly, avocado seeds were separated from the fruit and frozen in liquid nitrogen, followed by trituration. The powder obtained was extracted with hexane (C_6_H_14_, J.T. Baker) for 14 h in a Soxhlet apparatus. This lipid-rich fraction was filtered and then cooled to -18°C overnight for a cold crystallization. Precipitated crystals were recovered discarding the supernatant and then drying with nitrogen gas. This extract contains abundant molecules of 16-24 carbon aliphatic chains with hydroxyl groups such as aliphatic acetogenins and long-chain fatty acids as determined by gas chromatography-mass spectrometry (GC-MS) (Table 1). For biological assays, crystals were resuspended in dimethyl sulfoxide (DMSO 5%). In all of the experiments, the final concentration of DMSO was 0.1%.

### 2.2. Staphylococcus aureus Strain

The *S. aureus* subsp. *aureus* (ATCC27543) strain was used in this study. The strain was isolated from a case of bovine mastitis that has the capability to internalize into bMECs [3]. Bacteria were grown overnight in Luria-Bertani (LB) broth (Bioxon, México). For the different assays, the colony-forming units (CFU) were adjusted by measuring their optical density at 590 nm (OD 0.2 = 9.2 × 10^7^ CFU/ml).

### 2.3. Primary Culture Bovine Mammary Epithelial Cells (bMECs)

bMECs were isolated of alveolar tissue from the udder of a healthy lactating cow as described [17]. Cells from passages 2-8 were used in all of the experiments. The bMECs were cultured in growth medium (GM) that was composed of DMEM medium/nutrient mixture F12 Ham (DMEM/F12, Sigma) supplemented with 10% fetal bovine serum (Biowest), 10 *μ*g/ml insulin (Sigma), 5 mg/ml hydrocortisone (Sigma), 100 U/ml penicillin, 100 *μ*g/ml streptomycin, and 1 *μ*g/ml amphotericin B (Sigma). The cells were grown in a 5% CO_2_ atmosphere at 37°C.

### 2.4. Effect of Lipid-Rich Extract from Avocado Seed on S. aureus 27543 Growth and bMEC Viability

The effect of LEAS on *S. aureus* growth was determined by turbidimetry assay. For this, 9.2 × 10^7^ CFU/ml was cultured at 37°C in LB broth supplemented with different concentrations of LEAS (1-500 ng/ml) and growth was monitored turbidimetrically (590 nm) at 2, 6, 12, and 24 h.

To analyze the effect of LEAS on bMEC viability, 1 × 10^4^ cells were incubated with the extract (1-500 ng/ml) during 24-48 h at 37°C in 96-well flat-bottom plates in GM without supplements. Further, cells were detached with trypsin-EDTA (Sigma) and resuspended in a 1 : 1 dilution with 0.4% Trypan blue solution (Sigma) and incubated for 3 minutes. Finally, nonviable and viable cells were counted in a hemocytometer.

### 2.5. Effect of Lipid-Rich Extract from Avocado Seed on S. aureus 27543 Adhesion and Internalization into bMECs

Gentamicin protection assay was carried out using bMECs monolayers (~2 × 10^5^ cells) cultured in 24-well dishes treated with 6-10 *μ*g/cm^2^ rat tail type I collagen (Sigma) as described [3]. bMECs were incubated with different concentrations of LEAS (1-200 ng/ml) in DMEM/F12 media (Sigma) without supplements for 24 h followed by *S. aureus* infection (MOI 30 : 1 bacteria per cell). For this, the bMECs were inoculated with bacterial suspensions (9.2 × 10^7^ CFU/ml) and incubated for 2 h in 5% CO_2_ at 37°C. Afterward, bMECs were washed three times with PBS (pH 7.4) and incubated with DMEM medium supplemented with 80 *μ*g/ml gentamicin for 1 h at 37°C to eliminate extracellular bacteria. Finally, bMEC monolayers were detached with trypsin EDTA (Sigma) and lysed with 250 *μ*l of sterile distilled water. bMEC lysates were diluted 100-fold and plated on LB agar, and Petri dishes were incubated overnight at 37°C. The number of CFUs was determined by the standard colony counting technique. Data are presented as the percentage of internalization in relation to control (bMECs treated with vehicle).

To determine the adhesion of *S. aureus* on bMECs, cells were cultured and treated as described above but the gentamicin treatment was omitted. Data are presented as the percentage of adhesion in relation to control (bMECs treated with vehicle).

### 2.6. Staphylococcus aureus Viability in bMEC-Conditioned Media

To evaluate *S. aureus* survival in conditioned media, bMECs were cultured (~2 × 10^5^ cells) in 24-well plates with 6-10 *μ*g/cm^2^ rat tail type I collagen (Sigma). Then, bMECs were treated with LEAS (100 ng/ml) for 24 h and the culture medium was recovered. Next, conditioned media were inoculated with *S. aureus* suspension (9.2 × 10^7^ CFU/ml) and incubated for 2 h at 37°C and 180 rpm. Finally, a dilution of this suspension (1 : 1000) was plated on LB agar and incubated overnight at 37°C. The number of CFUs was determined by the standard colony counting technique.

### 2.7. Effects of Lipid-Rich Extract from Avocado Seed on MAP Kinase Activation in bMECs

The evaluation of the MAP kinase activation (p38, JNK, or ERK1/2) was carried out as reported [18]. Briefly, bMEC monolayers were cultivated on 96-well flat-bottom plates that were coated (Corning-Costar) with 6-10 *μ*g/cm^2^ rat tail type I collagen (Sigma). MAP kinase activation levels were assessed by flow cytometry in bMECs pretreated with LEAS (100 ng/ml, 24 h). pp38 (T180/Y182), pJNK1/2 (T183/185), and pERK1/2 (T202/Y204) were quantitatively determined using a Flex Set Cytometric Bead Array (Becton Dickinson) according to the manufacturer's protocol using a BD Accuri™ C6 flow cytometer. Data were analyzed with FCAP software (Becton Dickinson). A total of 3000 events were acquired following the supplied protocol. The minimum detection levels for each phosphoprotein were 0.38 U/ml for pJNK and 0.64 U/ml for pp38 and pERK. The corroboration of MAPK activation was performed using different inhibitors (data not shown).

### 2.8. RNA Isolation and Innate Immune Response Gene Expression Analysis

To analyze the effects of LEAS and/or *S. aureus* on the expression of IIR genes of bMECs, monolayers of cells cultured in 6-well plates with 6-10 *μ*g/cm^2^ rat tail type I collagen (Sigma) were incubated with LEAS 100 ng/ml (24 h) and/or *S. aureus* for 2 h (MOI 30 : 1). bMEC total RNA (5 *μ*g) was extracted with the TRIzol reagent (Invitrogen) according to the manufacturer's instructions. Genomic DNA contamination was removed from RNA samples with DNase I treatment (Invitrogen). Then, cDNA was synthesized as described [19]. The relative quantification of gene expression (qPCR) was performed using the comparative Ct method (ΔΔCt) in a StepOne Plus Real-Time PCR System (Applied Biosystems) according to the manufacturer's instructions. The reactions were carried out with VeriQuest SYBR Green qPCR Master Mix (Affymetrix). Specific primer pairs were acquired from Invitrogen and Elim Biopharm (Table 2), and their specificity was determined by endpoint PCR. The GAPDH gene was used as an internal control.

For the measurement of the TNF-*α* and IL-1*β* concentrations in the conditioned medium, bMECs were treated with LEAS (100 ng/ml) and/or *S. aureus*. The concentrations of TNF-*α* were measured using the DuoSet ELISA Development Kit (R&D Systems) according to the manufacturer's instructions, and the concentrations of IL-1*β* were assessed using the bovine IL-1*β* screening kit (Thermo Scientific).

### 2.9. Determination of Nitric Oxide (NO) and Reactive Oxygen Species (ROS)

Nitric oxide was estimated by Griess reaction. For this, bMECs were cultured (~2 × 10^5^ cells) in 24-well plates with 6-10 *μ*g/cm^2^ rat tail type I collagen (Sigma). Then, bMECs were treated with LEAS (100 ng/ml) for 24 h. After treatment, the cells were infected with *S. aureus* (2 h) and the conditioned medium was recovered. The NO secreted by bMECs into culture medium was evaluated by measuring the nitrite concentration (NO^2-^) in cell-free media using the Griess reaction as described [3].

To analyze the production of ROS, the method of Tarpey and Fridovich [20] was used. For this, bMECs (1 × 10^5^ cells) were grown in 24-well plates (Corning Costar) until 80% of confluence. Afterward, the LEAS (100 ng/ml) was added for 24 h; then, bMECs were infected with *S. aureus* (2 h). Subsequently, bMECs were detached with trypsin, recovered, and washed with PBS by centrifugation and the supernatant was removed. bMECs were incubated for 30 minutes with dihydrorhodamine-123 (DHR) 5 mM (Molecular Probes). ROS were determined by flow cytometry (BD Accuri™ C6 flow cytometer). In both evaluations, LPS was used as a positive control.

### 2.10. Data Analysis

The data were obtained from three independent experiments; each one was performed in triplicate and compared with one-way analysis of mean comparisons using Student's *t*-test, except for ELISA; in this case, a one-way analysis of variance (one-way ANOVA) using the Tukey-Kramer test was used. The results are reported as the means ± the standard errors (SE), and the significance level was set at *P* ≤ 0.05, except for RT-qPCR analysis where fold change values greater than 2 or less than 0.5 were considered as significant differentially expressed mRNAs [18]. The data were normalized to vehicle (DMSO 0.1%).

## 3. Results

### 3.1. The Lipid-Rich Extract from Avocado Seed Does Not Affect S. aureus Growth and bMEC Viability

**Figure 1:**
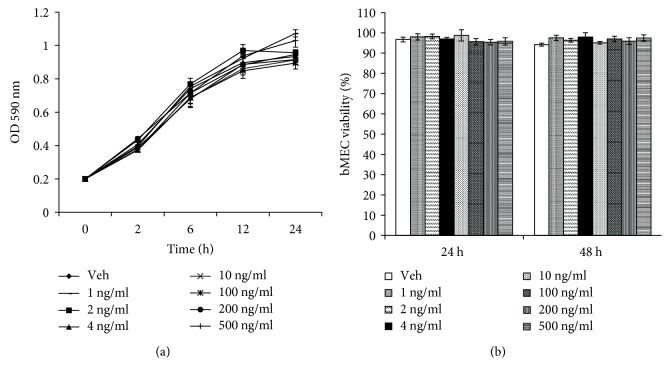
The lipid-rich avocado seed extract did not affect *S. aureus* growth and bMECs viability. (a) *S. aureus* was grown in LB at 37°C (18 h). The OD_590_ was adjusted at 0.2 (9.2 × 10^7^ CFU) and the treatments were added. The growth was monitored measuring the OD at the indicated time. (b) bMECs were grown in 96-well dishes (80% confluence) and the treatments were added. The viability was determined by Trypan blue exclusion assay at 24 and 48 h. The results correspond to three independent experiments performed by triplicate. Vehicle (DMSO 0.1%) (*P* ≤ 0.05, Student's *t*-test).

To evaluate the effect of LEAS on *S. aureus* growth, bacteria were cultivated in LB broth supplemented with different concentrations (1-500 ng/ml). The turbidimetric results showed that the *S. aureus* growth was not affected by LEAS at the times evaluated (Figure 1(a)). In the same way, the effect of LEAS on the viability of bMECs was evaluated at 24 and 48 h using the Trypan blue exclusion assay. Data showed that LEAS did not affect bMEC viability at any concentration tested (Figure 1(b)).

### 3.2. The Lipid-Rich Extract from Avocado Seed Inhibits the S. aureus Internalization into bMECs

**Figure 2:**
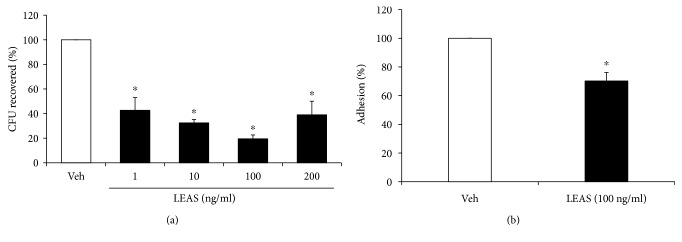
The lipid-rich avocado seed extract inhibits *S. aureus* internalization into bMECs and the adhesion. (a) The effect of LEAS on the internalization of *S. aureus* into bMECs is presented as the percentage of CFU recovered after the lysis of bMECs. The values were determined considering the vehicle (DMSO 0.1%) as 100% of internalization. The results are the average of three independent experiments performed in triplicate. Vehicle (DMSO 0.1%). ∗ indicates significant changes (*P* ≤ 0.05, Student's *t*-test). (b) To determine the adhesion of *S. aureus* on bMECs, cells were cultured and treated as described in Materials and Methods but the gentamicin treatment was omitted. Data are presented as the percentage of adhesion in relation to control (bMECs treated with vehicle). ∗ indicates significant changes (*P* ≤ 0.05, Student's *t*-test).

The effect of LEAS on *S. aureus* internalization into bMECs was evaluated by gentamicin protection assay. bMECs were treated with LEAS (1-200 ng/ml) 24 h before bacterial challenge. According to the CFUs recovered, the LEAS (1-200 ng/ml) significantly decreased *S. aureus* internalization into bMECs (60-80%) in all of the concentrations tested in relation to vehicle (Figure 2(a)). The more pronounced inhibitory effect was observed at 100 ng/ml (80%). Interestingly, this concentration also decreased the *S. aureus* adhesion to bMECs (~30%) (Figure 2(b)). According to these results, we used this concentration in the rest of the experiments.

### 3.3. The Lipid-Rich Extract from Avocado Seed Improves the Defense of bMECs

**Figure 3:**
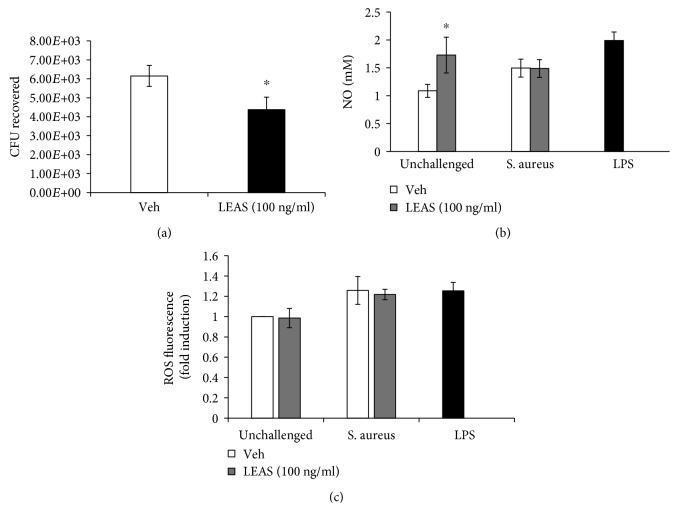
The conditioned media of LEAS-pretreated bMECs showed antimicrobial activity against *S. aureus*. (a) Effect of conditioned medium from bMECs treated with vehicle (DMSO 0.1%) or LEAS (100 ng/ml) for 24 h on *S. aureus* viability. Bacteria were incubated 2 h with conditioned media. The number of CFU recovered is shown. The results are the average of three independent experiments performed in triplicate. Vehicle (DMSO 0.1%). ∗ indicates significant changes (*P* ≤ 0.05, Student's *t*-test). (b) Nitric oxide and (c) ROS production in bMECs treated with 100 ng/ml of LEAS for 24 h and then challenged with *S. aureus* for 2 h. NO production was measured as NO^2-^ concentration in culture medium. ROS production was evaluated by flow cytometry. In (b, c), cells stimulated with LPS (1 *μ*g/ml, 24 h) were used as a positive control. Each bar shows the mean of triplicate ± SE of three independent experiments. ∗ indicates significant changes (*P* ≤ 0.05, Student's *t*-test) within the treatment. Vehicle (DMSO 0.1%).

In order to evaluate if the inhibitory effect of LEAS on *S. aureus* internalization into bMECs was related to the secretion of antimicrobial products by bMECs, we evaluated bacterial viability in the presence of conditioned media (culture media of bMECs treated 24 h with LEAS). Interestingly, bacterial viability diminished by ~30% in the presence of conditioned media, which suggests that LEAS induced the production and secretion of antimicrobial molecules in bMECs (Figure 3(a)).

Then, we evaluate if reactive oxygen and nitrogen species could contribute to the antimicrobial effect showed by the conditioned media. The results showed that bMECs treated with LEAS (100 ng/ml) for 24 h increased the NO levels; this effect was maintained even in bMECs challenged with *S. aureus* (Figure 3(b)). However, the ROS production was not affected by LEAS treatment (Figure 3(c)). These results suggest that NO production could be involved in the antimicrobial effect detected in the conditioned media.

### 3.4. The Lipid-Rich Extract from Avocado Seed Inhibits p38 but Not JNK1/2 or ERK1/2 in bMECs

**Figure 4:**
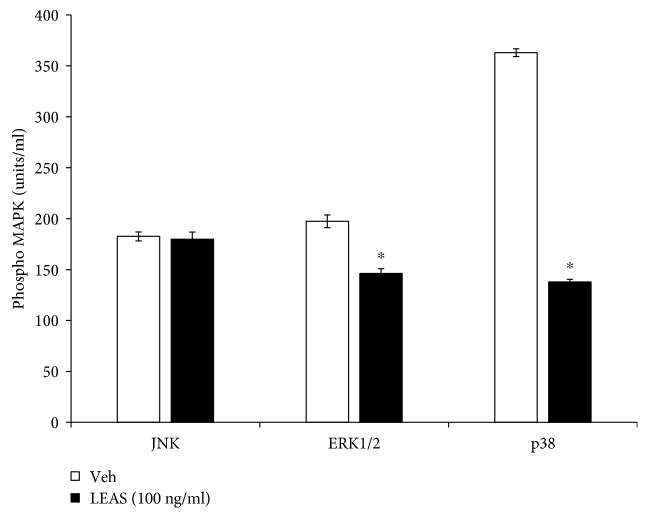
p38, JNK, and ERK1/2 activation regulated by LEAS in bMECs. MAPK phosphorylation was measured in bMECs that were treated with LEAS (100 ng/ml) by flow cytometry. Each bar shows the result of one experiment and a total of 3000 events were acquired. ∗ indicates significant changes (*P* ≤ 0.05, Student's *t*-test) within the treatment. Vehicle (DMSO 0.1%).

MAPK activation has been involved in *S. aureus* internalization into bMECs [18]. Thus, we evaluated whether LEAS regulates MAPK activation (p38, JNK, or ERK1/2) in bMECs. Interestingly, in LEAS-pretreated bMECs the basal activation of JNK and ERK1/2 was not modified; however, phosphorylated p38 was diminished by ∼60% (Figure 4).

### 3.5. The Lipid-Rich Extract from Avocado Seed Regulates the Expression of Innate Immune Elements in bMECs

bMECs are key elements of IIR and play a significant role in the defense against pathogens. Consequently, we analyzed the mRNA levels of the proinflammatory cytokines TNF-*α*, IL-1*β*, and IL-6 and the anti-inflammatory cytokine IL-10, as well as the antimicrobial peptides TAP, DEFB1, and BNBD5 in LEAS-pretreated bMECs before and after infection. LEAS favors an anti-inflammatory response in bMECs due to the decrease in the mRNA levels of the proinflammatory cytokine TNF-*α* (~0.5-fold) and the increase of IL-10 mRNA levels significantly (~11-fold). Notably, the effect on IL-10 was more pronounced in bMECs challenged with *S. aureus* (~22-fold) (Table 3). Furthermore, LEAS kept the reduction of the TNF-*α* secretion in the infected bMECs. Only the secretion of IL-1*β* was increased (~1.5-fold) when the LEAS-treated cells were infected with *S. aureus* (Table 4). Also, the mRNA levels for IL-1*β* and IL-6 were not modified for any of the conditions evaluated. In addition, we evaluated the mRNA levels of the antimicrobial peptides BNBD5, DEFB-1, and TAP. The treatment of bMECs with LEAS did not affect the mRNA expression of the antimicrobial peptides tested; however, the challenge with the bacteria increased the BNBD5 expression (~4-fold).

## 4. Discussion

IIR modulation is an alternative for therapeutic or prophylactic treatment to control and prevent diseases. Previously, we showed that short- and medium-chain fatty acids reduced *S. aureus* internalization into bMECs and improved IIR [3, 8]. In this sense, avocado (a fruit rich in fatty acids) is attractive in the search of plant compounds with immunomodulatory properties. This work demonstrates that a lipid-rich extract from Mexican avocado seed reduced *S. aureus* internalization into bMECs and regulated the IIR.

Long-chain fatty acids have antimicrobial activity against *S. aureus* [21]. Oleic acid and lauric acid (>70 *μ*g/ml) have showed antimicrobial effects against methicillin-resistant *S. aureus* [22]. Also, eicosapentaenoic acid and docosahexaenoic acid (128-256 mg/ml) have antimicrobial effects against *S. aureus* isolates from diverse origins, including isolates methicillin and vancomycin resistant [23]. Likewise, docosahexaenoic acid (30 *μ*M) induces apoptosis in breast cancer cell lines MCF-7 and SK-BR-3 [24]. Avocado seed extract is rich in fatty acids (mainly palmitic, oleic, and linoleic), and derivatives such as acetogenins, avocatins, persins, pahuatins, or fatty acid alcohols have antimicrobial and cytotoxic activities [14, 25]. Noteworthy, LEAS (1-500 ng/ml) did not affect the *S. aureus* growth and bMEC viability (Figure 1). It is important to notice that the concentrations used in this work are lower than those reported with antibacterial and cytotoxicity properties. However, preliminary results of our group indicate that LEAS are cytotoxic to cancer cells (Caco-2) at concentrations above of 10 *μ*g/ml (data unpublished).

Bacterial adhesion and internalization are important for the establishment of chronic bovine mastitis and thus are an attractive target in the implementation of strategies for its control. In a previous work, we demonstrated that pretreated bMECs with short- and medium-chain fatty acids showed a reduced *S. aureus* internalization. Sodium propionate (1-5 mM) decreased bacterial invasion by ~65%, whereas sodium butyrate (0.25 mM) inhibited it by ~50% [3]. Likewise, sodium hexanoate (0.25-5 mM) reduced *S. aureus* internalization by ~60% [26]. Similarly, LEAS inhibited *S. aureus* internalization into bMECs but its effect was more pronounced than those reported for short- and medium-chain fatty acids, reaching inhibition of 80% (Figure 2). Also, the inhibitory LEAS concentrations were lower suggesting a better effect of lipids from avocado seed. Additionally, LEAS diminished the *S. aureus* adhesion to bMECs (~30%), which partially explains the inhibited internalization by LEAS. Interestingly, in LEAS-pretreated bMECs, the levels of NO in conditioned media were increased and bacteria viability was reduced by ~30% when incubated with it (Figure 3(a)). This data suggests a participation of antimicrobial molecules in the reduction of bacterial internalization. This result agrees with a previous report, in which the inhibitory effect of estradiol on *S. aureus* internalization into bMECs was associated to the secretion of elements with antimicrobial activity in the conditioned media [27]. In the same way, de Lima et al. [28] reported that the NO production of macrophages was stimulated by fatty acids in a concentration-dependent manner; low concentrations (1-10 *μ*M) stimulated NO production in J774 cells (murine macrophages), whereas high concentrations (50-200 *μ*M) inhibited NO production. This data supports the fact that IIR of bMECs can be improved by immunomodulatory molecules which leads to the secretion of antimicrobial molecules that could contribute to the reduction in bacterial internalization.

On the other hand, for *S. aureus* internalization into bMECs, the MAPK pathway has a relevant role. When MAPK activity was blocked with pharmacological inhibitors of p38 (2.5–10 *μ*M SB203580), JNK (5–20 *μ*M, SP600125), or ERK1/2 (0.62–10 *μ*M, U0126), a considerable reduction in *S. aureus* internalization was observed, indicating that these kinases are involved in this process [18]. Interestingly, LEAS inhibited the activation of MAPK p38 ~60% (Figure 4), suggesting that this kinase could have a relevant participation in the observed reduction of internalization. However, other approaches are necessary to determine the precise participation of p38 in this process.

Mastitis is an inflammation of the mammary gland caused principally by bacteria, which leads to the activation of the innate immune system [29]. TNF-*α* is a rapid-response proinflammatory cytokine expressed in bMECs and plays an important role in mastitis. Bacterial stimulation of bMECs induces the expression of TNF-*α* but is depending of the strain [30, 31]. bMECs challenged with *S. aureus* induced the expression of TNF-*α* up to 11-fold (data not shown), which coincides with the reported by our group [26]. However, bMECs treated with vehicle (DMSO 0.1%) and challenged 2 h with bacteria maintained the basal expression of the TNF-*α* mRNA (Table 3), which was attributable to the anti-inflammatory effects of DMSO, as reported in Caco-2 cells [32]. Noteworthy, LEAS (100 ng/ml) treatment decreased the expression of this cytokine 0.5-fold in bMECs, which was maintained even when cells were infected. This result is in accordance with a downregulation of TNF-*α* in pretreated bMECs with cholecalciferol (a lipid molecule) [19]. Also, we detected similar levels of TNF-*α* secretion in bMECs infected or pretreated with LEAS before the *S. aureus* challenge. However, the IL-1*β* secretion was induced in the infected LEAS-treated cells (Table 4) but not the mRNA expression of this gene. This effect can be the consequence of the activation of other mechanisms, such as inflammasome activation, which requires further research [33]. Interestingly, pretreated bMECs with LEAS showed an upregulation in IL-10 expression, which was more pronounced in bMECs challenged with bacteria (~20-fold). These results are attractive due to the fact that this cytokine is not significantly induced by *S. aureus* in the udder [34]. Interestingly, these anti-inflammatory effects of LEAS on bMECs (TNF-*α* downregulation and IL-10 upregulation) correlated with the decrease in the internalization of *S. aureus* into bMECs.

With regard to antimicrobial peptides, these molecules actively contribute to IIR by direct action against microbial infection and as a component of the inflammatory response [35]. It has been reported that bMECs express diverse antimicrobial peptides: among them are the *β*-defensins. During IIR of the bovine mammary gland against bacterial infections, it has been reported that the expression of *β*-defensin and bovine neutrophil defensin 5 (BNBD5) increases significantly [3, 26]. Similarly, BNBD5 is expressed in the bovine mammary gland, especially in bMECs, and its expression levels depend on the bacterial stimulus [36]. However, in pretreated bMECs with LEAS, the expression of BNBD5 was not modified; only when these cells were challenged with *S. aureus*an increase was observed (Table 3). Nevertheless, it is necessary to evaluate other antimicrobial peptides in future experiments in order to determine their contribution in the *S. aureus* internalization reduction induced by LEAS.

According to the results of this work, we propose that LEAS could be applied as a prophylactic treatment to avoid bovine intramammary infections, because they improve bMEC innate immune response.

## 5. Conclusions

The results of this work shown that lipid-rich extract from avocado (LEAS) is a modulator of innate immune response in bovine mammary epithelial cells during *S. aureus* infection. Also, LEAS (100 ng/ml) inhibits bacterial internalization into bMECs. These data suggest that LEAS could be useful for mastitis control.

## Figures and Tables

**Table 1:** Composition of lipid-rich extract from Mexican avocado seed (*Persea americana* var. drymifolia).

Group	Compound	Content (*μ*g/g)
Fatty acid derivatives (aliphatic acetogenins)	Avocatins	32.28
	Persins	10.12
	Pahuatins	4.26
	Polyhydroxy fatty acids	24.26

Long-chain fatty acids	Myristic acid	2.49
	Palmitic acid	7.1
	Linoleic acid	4.06
	Oleic acid	5.32
	Stearic acid	5.06
	Arachidic acid	2.39
	Erucic acid	2.44
	Behenic acid	3.63
	Nervonic acid	2.88
	Tetracosanoic acid	4.29

**Table 2:** Bovine oligonucleotides used in this study.

Specificity	Primer	Sequence (5′-3′)	Fragment size (bp)	Annealing temperature (°C)	References
IL-1*β*	Forward	GCAGAAGGGAAGGGAAGAATGTAG	198	52	[19]
	Reverse	CAGGCTGGCTTTGAGTGAGTAGAA			

IL-6	Forward	AACCACTCCAGCCACAAACACT	179	57	[19]
	Reverse	GAATGCCCAGGAACTACCACAA			

TNF-*α*	Forward	CCCCTGGAGATAACCTCCCA	101	56	[19]
	Reverse	CAGACGGGAGACAGGAGAGC			

IL-10	Forward	GATGCGAGCACCCTGTCTGA	129	59	[19]
	Reverse	GCTGTGCAGTTGGTCCTTCATT			

BNBD5	Forward	GCCAGCTGAGGCTCCATC	143	55	[9]
	Reverse	TTGCCAGGGCACGAGATCG			

DEFB1	Forward	CCATCACCTGCTCCTCACA	185	54	[9]
	Reverse	ACCTCCACCTGCAGCATT			

TAP	Forward	GCGCTCCTCTTCCTGGTCCTG	216	57	[9]
	Reverse	GCACGTTCTGACTGGGCATTGA			

GAPDH	Forward	TCAACGGGAAGCTCACTGG	237	56.9	[9]
	Reverse	CCCCAGCATCGAAGGTAGA			

**Table 3:** Effect of lipid-rich extract from avocado seed on expression of innate immune elements of bMECs.

Activity	Gene	Vehicle	LEAS (100 ng/ml)	Vehicle+*S. aureus*	LEAS (100 ng/ml)+*S. aureus*
Pro-inflammatory	TNF-*α*	1 ± 0	0.48 ± 0.07^∗^	1.03 ± 0.25	0.40 ± 0.09^∗^
	IL-1*β*	1 ± 0	1.03 ± 0.15	0.86 ± 0.29	1.43 ± 0.55
	IL-6	1 ± 0	0.74 ± 0.33	0.78 ± 0.18	0.59 ± 0.13

Anti-inflammatory	IL-10	1 ± 0	11.39 ± 3.57^∗^	2.73 ± 1.46^∗^	21.39 ± 5.9^∗^

Antimicrobial peptide	BNBD5	1 ± 0	0.94 ± 0.16	0.98 ± 0.22	4.02 ± 0.32^∗^
	DEFB1	1 ± 0	0.98 ± 0.29	0.51 ± 0.23^∗^	0.35 ± 0.14^∗^
	TAP	1 ± 0	1.38 ± 0.29	0.44 ± 0.17^∗^	1.60 ± 0.34

Relative expression of genes was determined by RT-qPCR using GAPDH as endogenous control. The results correspond to three independent experiments and show the mean of triplicate ± SE. ^∗^Fold change values greater than 2 or less than 0.5 were considered significant differentially expressed mRNAs with respect to vehicle.

**Table 4:** Effect of lipid-rich extract from avocado seed on secretion of cytokines in bMECs.

Cytokine (pg/ml)	Vehicle	LEAS (100 ng/ml)	Vehicle+*S. aureus*	LEAS (100 ng/ml)+*S. aureus*
TNF-*α*	2459.83 ± 85.16^a^	2736.5 ± 72.85^a^	1614.83 ± 28.33^b^	1576.5 ± 123.92^b^
IL-1*β*	ND	ND	257.9 ± 24.76^b^	431.56 ± 38.02^a^

Proteins were determined by ELISA (pg/ml). The results correspond to three independent experiments and show the mean of triplicate ± SE. Different letters indicate significant differences within the row. ND: not detected.

## Data Availability

The data used to support the findings of this study are included within the article.
